# Role of IFN-γ and TGF-β in the Pathophysiology of Chronic Sinusitis -

**DOI:** 10.31661/gmj.vi.3948

**Published:** 2025-10-28

**Authors:** Amer Salih Khalaf, Ammar Mohammed Alwan, Amer Saleem Khalaf

**Affiliations:** ^1^ Tikrit University, College of Medicine, Department of Otorhinolaryngology, Salah Addin, Iraq

**Keywords:** Sinusitis, Rhinosinusitis, Allergy, Cytokines, TGF-beta, IFN-y

## Abstract

**Background:**

This study aimed to investigate the role of inflammatory cytokines IFN-γ and TGF-β in the pathophysiology of chronic sinusitis among Iraqi patients.

**Materials and Methods:**

A case-control study was conducted in Salah-Al-Din Governorate from March to July 2024. We enrolled 60 clinically diagnosed chronic sinusitis patients from Tikrit Teaching Hospital and 30 healthy controls. Serum levels of IFN-γ and TGF-β were measured using sandwich ELISA.

**Results:**

The patient cohort (55% male) showed highest prevalence in age groups 20 (37%), 21-30 (25%), and 31-40 years (23%). The most frequent symptoms were nasal obstruction (98%), nasal discharge (95%), and reduced smell sensation (90%). IFN-γ and TGF-β levels were significantly elevated in patients (16.45±7.01 pg/mL and 32.27±11.38 ng/mL, respectively) compared to controls (6.95±2.34 pg/mL and 22.18±7.66 ng/mL; P0.05). ROC analysis demonstrated IFN-γ›s stronger association with disease status (AUC 0.83) than TGF-β (AUC 0.68). A weak positive correlation was observed between the cytokines (r=0.277, P0.05).

**Conclusion:**

Our findings suggest IFN-γ and TGF-β play significant roles in the inflammatory processes of chronic sinusitis, particularly among younger patients. While both cytokines were elevated in patients, IFN-γ showed greater discriminatory potential. These results contribute to understanding the immunopathological mechanisms underlying chronic sinusitis.

## Introduction

Chronic sinusitis is characterized by persistent inflammation and swelling of the sinus mucous membranes, leading to symptoms such as pain, nasal obstruction, and periorbital or frontal edema. This condition might persist for at least 12 weeks despite appropriate treatment [1]. The resultant mucosal edema and impaired mucociliary clearance contribute to nasal congestion, while periocular swelling may also be observed. Etiologies include infectious pathogens (bacterial, viral, or fungal), nasal polyposis, or chronic mucosal inflammation. Chronic rhinosinusitis (CRS) encompasses a spectrum of clinical manifestations [2]. The condition affects both pediatric and adult populations, with a diagnostic threshold of ≥12 weeks of symptoms [3]. Risk factors include allergen exposure and recurrent upper respiratory infections. Allergic rhinitis, microbial pathogens, or fungal elements may perpetuate chronic inflammation, resulting in prolonged symptomatology lasting months to years [4]. According to Olufunsho and Akokhamen [5], interferon-gamma (IFN-γ) modulates inflammatory responses through leukocyte activation, natural killer (NK) cell stimulation, B-cell regulation, and eosinophil recruitment. IFN-γ is secreted by Th1 lymphocytes, CD8+ cytotoxic T cells, NK cells, and B cells, promoting a Th1-dominant immune microenvironment [6]. Histopathological studies demonstrate reduced IFN-γ protein expression in nasal polyp tissue compared to non-polypoid mucosa [7]. Despite low cytokine concentrations in homogenized tissue, flow cytometric analysis reveals a mixed Th1/Th2 inflammatory infiltrate, with a significant population of IFN-γ+ Th1 cells in nasal polyps [8].

Transforming growth factor-beta (TGF-β) is a critical immunoregulatory cytokine that maintains immune homeostasis and tolerance by suppressing proliferation and function of multiple immune cell lineages. TGF-β also exhibits pro-fibrotic activity. Cellular sources of TGF-β1 include fibroblasts, eosinophils, macrophages, and regulatory T (Treg) cells [9]. Prior research indicates impaired TGF-β signaling in chronic rhinosinusitis without nasal polyps (CRSsNP) [10]. Recent evidence suggests elevated TGF-β1 levels in patients with comorbid asthma or allergic conditions compared to healthy controls [10]. As evidence tells that IFN-γ plays a crucial role in Th1-mediated inflammation and leukocyte activation in chronic sinusitis [5][6], while TGF-β contributes to immune regulation and fibrosis [9][10], existing data is low regarding their combined role and diagnostic potential in Iraqi patients. Previous studies have shown dysregulated cytokine profiles in chronic sinusitis [7][8], but regional variations in immune responses necessitate population-specific investigations. We aimed at investigating the serum levels of IFN-γ and TGF-β in chronic sinusitis patients to clarify their pathophysiological contributions.

## Material and Methods

### Samples Collection

This study was conducted in Salah-Al-Din Governorate between March and July 2024. A total of 60 blood samples were collected from chronic sinusitis patients attending the outpatient clinic at Tikrit Teaching Hospital. All participants had been previously diagnosed by a physician. An additional 30 blood samples were obtained from healthy individuals with no history of sinusitis or respiratory infections in the past six months, serving as the age- and sex-matched control group. The inclusion criteria for patients required adults aged 18-65 years with a confirmed diagnosis of chronic sinusitis (symptoms persisting for >12 weeks) and no recent use of antibiotics, corticosteroids, or immunosuppressive drugs. Exclusion criteria included autoimmune diseases, malignancies, acute infections, pregnancy, lactation, or immunodeficiency disorders. The study protocol was approved by the Ethics Committee at Tikrit University, College of Medicine (Approval No. 7/63/557, dated February 19, 2024).

For the sample size calculation, we used a two-sided test with a Type I error rate (α) of 0.05 and a power (1−β) of 0.8, based on data from Van Bruaene et al. (2009) [11], where we recalculated the standard deviation (SD=12.16) from reported picogram and interquartile ranges to nanograms, with expected means of 30.76 and 39.81 for the first and second groups, respectively, and a 1:1 sample size ratio, yielding 29 participants per group and a total sample size of 58.

### Data and Sample Collection

A structured questionnaire was administered to collect demographic data (age, sex), clinical symptoms (nasal obstruction, nasal discharge, facial pain, headache), duration of illness, and previous treatments. For laboratory analysis, 5 mL of venous blood was drawn from each participant under aseptic conditions and collected in gel-activated serum separator tubes. The samples were centrifuged at 5000 rpm for 4 minutes to separate serum, which was then aliquoted into sterile Eppendorf tubes and stored at -80°C until analysis. Serum concentrations of IFN-γ (Interferon-gamma) and TGF-β (Transforming Growth Factor-beta) were quantified using Sandwich Enzyme-Linked Immunosorbent Assay (ELISA) kits (CUSABIO, China) following the manufacturer’s protocol. The ELISA procedure involved coating microplate wells with specific antibodies, adding serum samples and standards in duplicate, incubating at 37°C for 1 hour, washing to remove unbound substances, adding HRP-conjugated detection antibodies, developing color with TMB substrate, and measuring absorbance at 450 nm using a microplate reader (BioTek, USA).

### Statistical Analysis

Statistical analysis was performed using SPSS v.21.0 and GraphPad Prism v.10. Continuous variables (IFN-γ and TGF-β levels) were expressed as mean ± standard deviation (SD), while categorical variables (demographic and clinical characteristics) were presented as frequencies and percentages. Student’s t-test was used to compare serum levels of IFN-γ and TGF-β between patients and controls, and Pearson’s Chi-square test assessed associations between clinical characteristics. Receiver Operating Characteristic (ROC) curve analysis determined the area under the curve (AUC), cut-off values, sensitivity, specificity, and odds ratio (OR). Pearson’s correlation coefficient evaluated the relationship between IFN-γ and TGF-β levels, with a P-value ≤ 0.05 considered statistically significant.

## Results

**Table:** Table1. Frequencies and Percentages of Anthropometric Features of Chronic Sinusitis Patients and Healthy Controls

		cases		control		P-value
		n	%	n	%	
	≤20	15	25	7	23.33	
	21-30	19	31.67	9	30	
Age groups (years) (range; 7-65 years)	31-40	17	28.33	8	26.67	0.971
	41-50	3	5	2	6.66	
	51-60	4	6.67	2	6.66	
	>60	2	3.33	2	6.66	
Gender	Males	33	55	16	53.33	0.990
	Females	27	45	14	46.67	

**Table:** Table 2. Frequencies and Percentages of Symptoms of Chronic Sinusitis Patients

		N	%
	Nasal obstruction	59	98[a1] .33%
	Nasal discharge	57	95%
	Reduced smell sensation	54	90%
Symptoms	Facial pain	46	76[a2] .67%
	Halitosis	36	60%
	Allergic symptoms	25	41.67%
	Headache	21	35%
	Ear pain	17	28.33%

**Table:** Table3. Comparative Concentrations of IFN-y and TGF-Beta between Chronic Sinusitis Patients Versus Healthy

	Groups	N	Mean	SD	P value
IFN-y (pg/mL)	Patients	60	16.45	7.01	P<0.01
Healthy	30	6.95	2.34
TGF-Beta (ng/mL)	Patients	60	32.27	11.38	P<0.0
Healthy	30	22.18	7.66

**Table:** Table4. ROC Curve, Sensitivity, Specificity, and Odd Ratio of IFN-y and TGF-Beta Indicators in Screening Chronic Sinusitis Diseases

Variables	AUC	Std. Error	P value	cut off	Sn. %	Sp. %	Odd ratio (C.I.)
IFN-y (pg/mL)	0.842	0.069	P<0.01**	8.50	81	77	13.39 (6.81 to 26.33)
TGF-Beta (ng/mL)	0.677	0.085	P<0.05*	26.03	59	64	2.66 (1.50 to 4.72)

**Figure-1:**
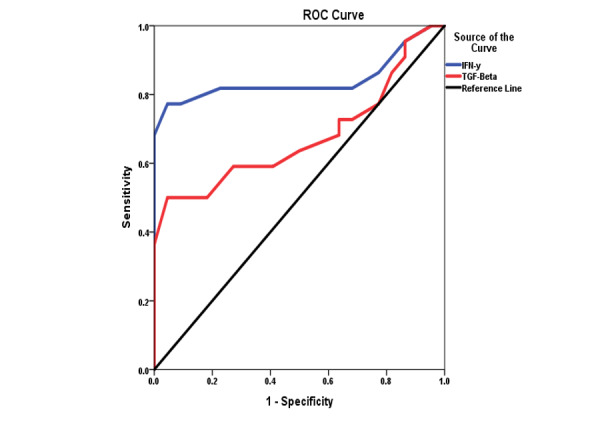
Figure 1. ROC cure of immunological markers in screening chronic sinusitis patients

Table-1 presents the frequencies and percentages of anthropometric features, including age groups and gender, among chronic sinusitis patients (cases) and healthy controls, showing no significant differences between the two groups (P>0.05 for all comparisons). The age distribution was similar, with the majority of participants in both groups falling within the 21-30 age range (cases: 31.67%, controls: 30%), while gender distribution was nearly equal (cases: 55% males, 45% females; controls: 53.33% males, 46.67% females), with P-values of 0.971 and 0.990 for age and gender, respectively, indicating no statistically significant differences.

Table-2 displays the frequencies and percentages of symptoms among chronic sinusitis patients, revealing significant differences (P<0.05) in their prevalence. Nasal obstruction was the most common symptom (98.33%), followed closely by nasal discharge (95%) and reduced smell sensation (90%). Facial pain was reported in 76.67% of cases, while halitosis (60%), allergic symptoms (41.67%), headache (35%), and ear pain (28.33%) were less frequent, indicating varying symptom burdens in chronic sinusitis. Pearson coefficient test showed there is no correlation between IFN-y and TGF-Beta (r=0.277; P=0.211). Table-3 compares the concentrations of IFN-γ and TGF-β between chronic sinusitis patients and healthy controls, showing highly significant differences (P<0.01). Patients had significantly higher mean levels of IFN-γ (16.45 ± 7.01 pg/mL) compared to controls (6.95 ± 2.34 pg/mL). Similarly, TGF-β levels were elevated in patients (32.27 ± 11.38 ng/mL) versus controls (22.18 ± 7.66 ng/mL), indicating a strong association between these cytokines and chronic sinusitis. Both differences were statistically highly significant (P<0.0).

### Receiver Operating Characteristic (ROC) Curve of Cytokines

Table-4 presents the diagnostic performance of IFN-γ and TGF-β in screening for chronic sinusitis using ROC curve analysis. IFN-γ demonstrated high predictive accuracy with an AUC of 0.842 (P<0.01), a cutoff value of 8.50 pg/mL, 81% sensitivity, 77% specificity, and a strong odds ratio of 13.39 (95% CI: 6.81-26.33). In contrast, TGF-β showed moderate diagnostic utility with an AUC of 0.677 (P<0.05), a cutoff of 26.03 ng/mL, 59% sensitivity, 64% specificity, and a lower odds ratio of 2.66 (95% CI: 1.50-4.72), as shown in Figure-1.

## Discussion

The current study aimed to investigate the role of inflammatory cytokines, particularly IFN-γ and TGF-β, in the pathophysiology of chronic sinusitis among Iraqi patients. Our findings revealed that the majority of sinusitis patients were males, with the highest prevalence in the <20 age group (37%), followed by 21-30 (25%) and 31-40 years (23%). These results align with previous studies, such as Khan and Saad [12], who reported a higher incidence of sinusitis in males aged 5-20 years. Similarly, Shaikh et al. [13] found that 61% of pediatric sinusitis cases occurred in the 5-10 age range, with a male predominance (54%). The increased susceptibility of children to sinusitis may be attributed to developmental factors, smaller sinus ostia, and frequent upper respiratory infections, which can lead to mucosal swelling and sinus obstruction [14].

Clinically, the most frequent symptoms observed in our cohort were nasal obstruction (98%), nasal discharge (95%), and reduced smell sensation (90%). These findings are consistent with prior research indicating that post-nasal drip and olfactory dysfunction are hallmark features of chronic sinusitis [15]. Khan and Saad [12] similarly reported that nasal discharge and obstruction were predominant symptoms, often resulting from allergic or microbial-induced inflammation. Furthermore, our observation of diminished olfactory function aligns with Lin and Yeh [16], who noted that over 60% of chronic rhinosinusitis patients experience smell impairment, likely due to combined inflammatory and anatomical factors. Olfactory dysfunction significantly impacts quality of life, contributing to emotional distress and reduced environmental awareness, underscoring the need for clinical assessment and targeted treatment [15].

At the molecular level, our study demonstrated significantly elevated serum levels of IFN-γ (16.45 ± 7.01 pg/mL) and TGF-β (32.27 ± 11.38 ng/mL) in patients compared to controls (6.95 ± 2.34 pg/mL and 22.18 ± 7.66 ng/mL, respectively; *P*<0.05). The pro-inflammatory role of IFN-γ in sinusitis was further supported by He et al. [17], who observed higher IFN-γ levels in rhinitis patients, correlating with disease severity. This cytokine is known to enhance eosinophil recruitment and sustain inflammatory responses [7]. Interestingly, reduced IFN-γ production in chronic rhinosinusitis (CRS) may impair antiviral defenses, suggesting that therapies like macrolides and glucocorticoids could restore interferon-mediated immunity [18].

In contrast, our data showed elevated TGF-β levels in sinusitis patients, differing from Carsuzaa et al. [9], who reported decreased TGF-β in nasal polyps. This discrepancy may reflect TGF-β’s dual role—acting as both an anti-inflammatory mediator (by suppressing IgE and eosinophil activity) [20] and a pro-fibrotic factor in tissue remodeling [23]. While TGF-β downregulation has been linked to nasal polyp formation and edema [9], its overexpression may contribute to epithelial-mesenchymal transition and fibrosis in chronic sinusitis [22]. These opposing effects highlight the complex interplay of TGF-β in sinusitis pathogenesis.

ROC analysis identified IFN-γ as a stronger diagnostic marker (AUC 0.83) than TGF-β (AUC 0.68), supported by its higher sensitivity, specificity, and predictive value. The weak correlation (r=0.277) between the cytokines suggests distinct pathways in disease progression.

While our study demonstrated elevated serum TGF-β levels in chronic sinusitis patients, Van Bruaene et al. [11] revealed divergent TGF-β signaling patterns in sinonasal tissue subtypes. Their work identified increased TGF-β1 protein, receptor (RI/RIII) expression, and collagen deposition in CRS without nasal polyps (CRSsNP), contrasting with reduced TGF-β1 and impaired signaling in CRS with polyps (CRSwNP). These tissue-specific findings complement our systemic cytokine measurements, suggesting that TGF-β’s role varies by disease endotype: it may drive fibrotic remodeling in CRSsNP (consistent with our observed elevations) but is suppressed in CRSwNP, aligning with Carsuzaa et al.’s [9] reports of low TGF-β in polyps. Notably, Van Bruaene et al. [11] linked these differences to collagen dysregulation, excessive in CRSsNP and deficient in CRSwNP, highlighting TGF-β’s dual role in inflammation and tissue remodeling. Our study extends these insights by demonstrating IFN-γ’s superior diagnostic value over TGF-β, even amid TGF-β’s context-dependent fluctuations.

## Conclusion

Our study highlights that younger individuals (10-40 years) are at higher risk for chronic sinusitis, with nasal obstruction, discharge, and olfactory dysfunction as key symptoms. Elevated IFN-γ and TGF-β levels underscore their roles in inflammation, though IFN-γ demonstrates superior diagnostic utility. Further research is needed to clarify their mechanistic contributions and therapeutic potential.

## Conflict of Interest

None.
